# Development and psychometric validation of the AI chatbots acceptance and perception scale for higher education students

**DOI:** 10.1038/s41598-026-62798-4

**Published:** 2026-07-22

**Authors:** Ashraf Ragab Ibrahim, Mohamed Ali Nemt-allah

**Affiliations:** Educational Psychology and Statistics Department, Faculty of Education, Al-Azhar University, Dakahlia, Egypt

**Keywords:** Artificial Intelligence chatbots, Higher education, Scale validation, Psychometric assessment, Mathematics and computing, Psychology, Psychology

## Abstract

The rapid integration of artificial intelligence (AI) chatbots in higher education has transformed student learning experiences and institutional operations, yet comprehensive measurement tools for assessing student usage patterns remain limited. This study presents the development and psychometric validation of the AI Chatbots Usage Scale, a comprehensive instrument measuring perceptual and attitudinal dimensions of AI chatbot acceptance among higher education students. Using a two-phase methodology, the research employed separate samples for Exploratory Factor Analysis (EFA) (*n* = 374) and Confirmatory Factor Analysis (CFA) (*n* = 599) among university students aged 17–23 from the Faculty of Education in Egypt. The initial 25-item scale underwent rigorous expert validation and pilot testing before final administration. EFA revealed a four-factor structure comprising Ease of Use, Perceived Usefulness, Trust, and Accessibility, accounting for 42.48% of total variance. CFA demonstrated excellent model fit indices, with the multidimensional model significantly outperforming a unidimensional alternative. Internal consistency reliability was excellent across all factors, with coefficients ranging from 0.731 to 0.928. The validated scale demonstrates strong theoretical alignment with established technology acceptance frameworks while extending traditional models to accommodate AI-specific considerations. These findings provide researchers and educational institutions with a reliable, theoretically grounded instrument for assessing and optimizing AI chatbot implementation in higher education contexts.

## Introduction

The contemporary educational landscape has witnessed an unprecedented transformation by integrating artificial intelligence technologies, contributing to shifts in how some institutions deliver instruction, support services, and administrative functions. The current landscape of AI technology adoption in academic institutions demonstrates rapid expansion across multiple domains, with particularly strong integration in personalized learning platforms, intelligent tutoring systems, and administrative functions^1–4^. This technological revolution has created new educational delivery paradigms while presenting complex challenges related to implementation, accessibility, and educational equity.

Despite the promising potential of AI integration in higher education, adoption patterns reveal significant disparities that reflect broader global inequalities in technological access and institutional capacity. These implementation efforts demonstrate notable variations across geographical regions, academic disciplines, and institutional readiness levels, with high-income countries and STEM fields leading implementation efforts while low-income regions face substantial infrastructural and financial barriers^5–8^. Such disparities highlight the critical need for comprehensive understanding of AI adoption patterns to ensure equitable access to technological innovations across diverse educational contexts.

Among the various AI applications in higher education, chatbots have emerged as particularly transformative tools that bridge the gap between technological capability and practical educational needs. The proliferation of AI chatbots across university platforms has accelerated dramatically, particularly following the introduction of advanced language models like ChatGPT^9,10^. This acceleration represents more than mere technological adoption; it signifies a fundamental shift in how educational institutions conceptualize student support, administrative efficiency, and learning facilitation.

The deployment of intelligent chatbot systems across diverse institutional functions has become increasingly sophisticated and comprehensive, encompassing student support services, administrative assistance, admissions processes, academic advising, library services, and virtual teaching assistant roles^11–13^. Universities worldwide have embraced chatbot technology to provide 24/7 support, streamline administrative processes, and enhance personalized learning experiences for students^14,15^. This widespread adoption reflects institutions’ growing recognition of chatbots’ potential to improve operational efficiency while meeting the evolving digital expectations of contemporary higher education students^16,17^.

The strategic investment in AI-powered educational technologies represents a significant commitment by institutions to modernize their pedagogical and administrative approaches. Educational institutions worldwide are increasingly investing in AI-powered technologies, including chatbots, intelligent tutoring systems, and adaptive learning platforms, to enhance teaching effectiveness and personalize learning experiences for diverse student populations^3,18^. These institutional investments require substantial infrastructure development, comprehensive training programs, and robust policy frameworks to ensure effective AI integration across academic settings^19^. The growing adoption of AI educational technologies reflects institutions’ commitment to modernizing pedagogical approaches and improving administrative efficiency through automated systems^4,20^. However, successful implementation depends heavily on institutional support and strategic planning to maximize educational benefits while addressing technological challenges^21^.

Understanding how students interact with AI chatbots has become crucial for optimizing these technological investments and ensuring positive educational outcomes. Students demonstrate diverse interaction patterns with AI chatbots, ranging from AI-reliant behaviors where chatbots serve as primary information sources to AI-collaborative approaches involving joint problem-solving activities^22,23^. Research indicates that training students in effective AI prompting skills significantly improves the quality of student-chatbot interactions and enhances learning outcomes across various academic disciplines^10,24^. While AI chatbots provide personalized learning experiences, immediate feedback, and increased student motivation, concerns exist regarding potential over-reliance and diminished independent problem-solving capabilities without proper guidance^25,26^. These varied interaction profiles underscore the importance of developing validated measurement tools to assess student AI chatbot usage patterns effectively.

The academic support capabilities of AI chatbots have positioned them as essential tools for enhancing student learning experiences across multiple dimensions of educational engagement. AI chatbots have emerged as transformative tools in higher education, serving multifaceted academic support functions that enhance student learning experiences. These intelligent systems primarily function as virtual tutors, providing personalized explanations, answering student queries, and guiding learners through complex assignments with immediate, individualized support^10,27,28^. Research demonstrates that AI chatbots effectively handle frequently asked questions, deliver instant feedback, and clarify intricate concepts, thereby supporting both self-paced and collaborative learning environments^29–32^.

Beyond academic support, AI chatbots have revolutionized administrative efficiency within higher education institutions, creating more streamlined and responsive institutional processes. AI chatbots have demonstrated significant utility in streamlining administrative services within higher education institutions, fundamentally transforming how students interact with institutional processes. These intelligent systems effectively automate routine administrative tasks including enrollment procedures, scheduling management, and information retrieval services, thereby reducing staff workload while simultaneously improving response times for student inquiries^10,33^. The implementation of chatbots in administrative contexts has proven particularly valuable in managing frequently asked questions and providing instant access to institutional information, creating more efficient operational frameworks that benefit both students and administrative personnel^27,34^.

The expansion of AI chatbots into comprehensive student services represents another critical advancement in educational technology application, addressing the holistic needs of university students beyond mere academic instruction. Student services represent another critical domain where AI chatbots have shown remarkable effectiveness in enhancing the overall educational experience. These systems provide comprehensive 24/7 support for student queries, offering accessible counseling support, career guidance, and campus navigation assistance that significantly reduces student stress and improves service accessibility^10,35^. The continuous availability of chatbot-mediated student services ensures that learners can access essential support resources regardless of time constraints, thereby promoting better academic outcomes and student satisfaction^20,28,32^.

The sophisticated capabilities of AI chatbots as research and learning enhancement tools have further expanded their educational utility, positioning them as comprehensive academic companions rather than simple information providers. Research and learning enhancement tools powered by AI chatbots have emerged as transformative educational resources that support personalized learning experiences and academic development. These systems function as virtual teaching assistants, delivering tailored feedback, supporting self-paced study methodologies, and adapting to individual learning needs while facilitating skill development across various disciplines^22,29,31^. Furthermore, chatbots provide valuable research assistance through literature summarization, idea generation, and data analysis support, while simultaneously offering automated assessment tools and formative feedback mechanisms that enable continuous progress tracking^12,36,37^.

Current theoretical frameworks explaining student acceptance and adoption of AI chatbots in higher education predominantly center on technology acceptance and innovation diffusion models. The Technology Acceptance Model (TAM) and its extensions (TAM3) remain foundational, emphasizing perceived usefulness, ease of use, and behavioral intention as primary determinants^38–40^. The Unified Theory of Acceptance and Use of Technology (UTAUT/UTAUT2) integrates multiple acceptance constructs, incorporating performance expectancy, effort expectancy, social influence, and facilitating conditions^41–43^. Additionally, the Diffusion of Innovation Theory examines relative advantage, compatibility, and trialability as adoption predictors^44^. These frameworks collectively inform comprehensive measurement instrument development by specifying key constructs, guiding operationalization through structural equation modeling, and ensuring contextual relevance for educational settings^17,45,46^.

Several measurement instruments have been developed to assess student interactions with AI chatbots; however, each presents notable limitations that the current study seeks to address. Köhler and Hartig^47^ developed instruments assessing student knowledge, usage, and attitude toward ChatGPT, yet their scale was platform-specific and not grounded in a comprehensive technology acceptance framework. Çobanoğullari and Özbek^48^ developed a ChatGPT usage scale for foreign language learners, limiting its applicability to a single disciplinary context and a specific student population. Nemt-allah et al.^49^ validated a ChatGPT Usage Scale among postgraduate students, again restricting generalizability across broader undergraduate populations and AI chatbot platforms beyond ChatGPT. In contrast, the present study addresses these gaps by developing a platform-agnostic, theoretically grounded instrument applicable across disciplines and undergraduate populations, explicitly integrating established technology acceptance frameworks within a single psychometrically validated multidimensional structure.

Building on these frameworks, the present scale focuses on four theoretically motivated constructs. Ease of Use refers to the degree to which students perceive AI chatbots as requiring minimal cognitive effort^41,50^. Perceived Usefulness reflects students’ beliefs that chatbot use enhances academic performance^51,52^. Trust captures students’ confidence in the accuracy, reliability, and integrity of AI-generated responses — a construct uniquely salient for AI systems that rely on opaque algorithmic processes^45,46^. Accessibility reflects the availability of technical infrastructure and institutional support enabling chatbot use, corresponding to UTAUT’s “facilitating conditions”^41–43^. These four constructs were prioritized over others such as social influence and hedonic motivation (enjoyment) because they represent core adoption barriers most reported in educational chatbot research^17,45,46^, and because our study focused on core acceptance barriers rather than affective or social dimensions.

Given the rapid expansion of AI chatbot implementation in higher education and the diverse ways students interact with these systems, there exists a critical need for validated measurement instruments that can accurately assess usage patterns, effectiveness, and impact on student learning outcomes. Despite the growing body of research on AI chatbots in educational settings, there remains a significant gap in psychometrically validated scales specifically designed to measure university students’ perceptions, attitudes, and acceptance of AI chatbots comprehensively. The development of such measurement tools is essential for researchers, educators, and institutions seeking to understand, evaluate, and optimize the integration of AI chatbots in higher education contexts. Therefore, this study aims to develop and psychometrically validate a comprehensive AI Chatbots Usage Scale specifically designed for higher education students, providing researchers and institutions with a reliable instrument to assess and understand student engagement with these transformative educational technologies.

## Method

### Research design

This study employed a cross-sectional survey design with a psychometric validation approach to develop and validate the AI Chatbots Usage Scale for university students. The research was conducted during the 2024–2025 academic year and utilized a two-phase methodology involving separate samples for exploratory and confirmatory analyses.

### Participants

Two distinct samples were recruited from the Faculty of Education, Tafhna Al-Ashraf, Dakahlia Governorate, Egypt, to conduct separate scale development and validation analyses. The first sample, designated for EFA, comprised 374 university students aged 17 to 23 (*M* = 18.52, *SD* = 1.23). The second sample, utilized for CFA, consisted of 599 university students aged 17 to 23 (*M* = 18.58, *SD* = 1.31). All participants were active users of artificial intelligence chatbots, ensuring their familiarity with the technology under investigation. The recruitment of two separate samples followed established psychometric practices to avoid capitalization on chance and to validate the scale structure robustly.

### Instruments

The AI Chatbots Usage Scale was developed as a 25-item instrument to measure university students’ usage patterns and experiences with artificial intelligence chatbots. The scale utilized a five-point Likert response format ranging from “Always” to “Never, " providing participants with sufficient response variability to reflect their usage behaviors and attitudes accurately. The initial version of the scale underwent rigorous expert validation to ensure content validity and clarity. Nine subject matter experts in educational psychology and artificial intelligence applications reviewed the preliminary scale items. These experts assessed each item for clarity, relevance, and appropriateness for the target population of university students. Based on their comprehensive feedback, three items were removed from the scale due to concerns regarding clarity and conceptual overlap with other items. The expert review process ensured that the remaining items adequately represented the construct domain while maintaining linguistic and cultural appropriateness for Arabic-speaking university students.

Following expert review, the scale was administered to a pilot sample of 48 university students to evaluate item clarity and comprehensibility from the student perspective. This pilot testing phase allowed for identifying ambiguous wording or confusing instructions that might affect response quality. Based on student feedback regarding item interpretation and response difficulty, necessary modifications were implemented to enhance the scale’s accessibility and user-friendliness. The pilot testing ensured that the target demographic would understand the final version of the scale and elicit meaningful responses.

A rigorous translation process was implemented, given the bilingual nature of academic research and the need for international applicability. The scale was originally developed in Arabic, the native language of the participants, to ensure cultural and linguistic appropriateness. Subsequently, three independent specialists conducted forward translations from Arabic to English, ensuring conceptual equivalence rather than literal translation. Two additional specialists performed back-translation from English to Arabic to verify translation accuracy and maintain semantic integrity.

### Data collection procedures

Data collection was conducted entirely digitally using Google Forms, which provided several advantages, including standardized presentation, automatic data compilation, and accessibility for geographically dispersed participants. The online format was particularly appropriate given that the scale measures digital technology usage, creating consistency between the measurement tool and the assessed construct. Participants accessed the survey through institutional channels and completed it voluntarily. The digital collection method ensured data integrity by preventing incomplete submissions and reducing transcription errors commonly associated with paper-based surveys.

### Data analysis strategy

Statistical analyses were performed using two complementary software packages to evaluate the scale’s psychometric properties comprehensively. SPSS version 27 was utilized for descriptive statistics, reliability analyses, and EFA procedures. AMOS version 26 was employed for CFA to test the proposed factor structure and evaluate model fit indices. This dual-software approach provided robust analytical capabilities, allowing for thorough examination of the scale’s factor structure, internal consistency, and construct validity.

## Results

### Exploratory factor analysis

The Kaiser-Meyer-Olkin (KMO) measure of sampling adequacy yielded a value of 0.918, indicating excellent suitability of the data for factor analysis. Bartlett’s test of sphericity was statistically significant (χ² = 2685.570, df = 231, *p* < .001), confirming that the correlation matrix was not an identity matrix and that factor analysis was appropriate. These preliminary analyses supported proceeding with EFA to examine the underlying factor structure of the AI Chatbots Usage Scale.

Principal axis factoring with Promax rotation was conducted on the 22-item scale to explore the factor structure. Factor retention was determined using a combination of parallel analysis, scree plot inspection, and interpretability criteria. The analysis revealed a multidimensional solution with four factors, collectively accounting for 42.48% of the total variance. The first factor (Ease of Use) explained 29.31% of the variance with an eigenvalue of 6.448, followed by the second factor (Perceived Usefulness) explaining 6.02% with an eigenvalue of 1.324, the third factor (Trust) explaining 4.45% with an eigenvalue of 0.978, and the fourth factor (Accessibility) explaining 2.71% with an eigenvalue of 0.595. The retention of all four factors was supported by parallel analysis results and the interpretability of the factor solution, despite the third and fourth factors yielding eigenvalues below the conventional threshold of 1.0.

Examination of the factor loadings revealed that items loaded cleanly on their respective factors, with most loadings exceeding 0.50 (see Table 1). The Ease of Use factor contained eight items with loadings ranging from 0.561 to 0.719, the Perceived Usefulness factor included four items with loadings from 0.548 to 0.722, the Trust factor comprised four items with loadings between 0.544 and 0.648, and the Accessibility factor contained three items with loadings from 0.555 to 0.677. A competing unidimensional model was also examined, yielding a single factor that explained 29.31% of the variance, though the multidimensional solution demonstrated superior interpretability and theoretical coherence.

**Table 1:** Factor loadings for multidimensional and unidimensional models of the AI Chatbots Usage Scale.

Items	Multidimensional Model				Unidimensional Model
	Ease of Use	Perceived Usefulness	Trust	Accessibility	
12.	0.719				0.669
14.	0.672				0.602
15.	0.669				0.641
17.	0.641				0.601
16.	0.631				0.554
11.	0.598				0.574
13.	0.570				0.539
18.	0.561				0.591
4.		0.722			0.544
3.		0.707			
2.		0.578			0.597
1.		0.548			0.587
5.		.			0.591
6.					0.577
7.			0.648		0.533
8.			0.633		0.571
10.			0.574		0.564
9.			0.544		0.569
21.				0.677	
20				0.616	
22.				0.555	
19.					

### Confirmatory factor analysis

**Fig. 1:**
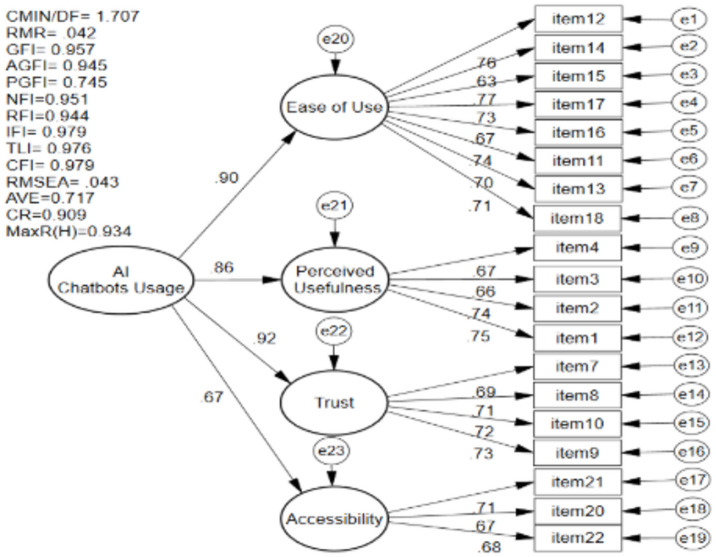
CFA path diagram for multidimensional four-factor model.

**Fig. 2:**
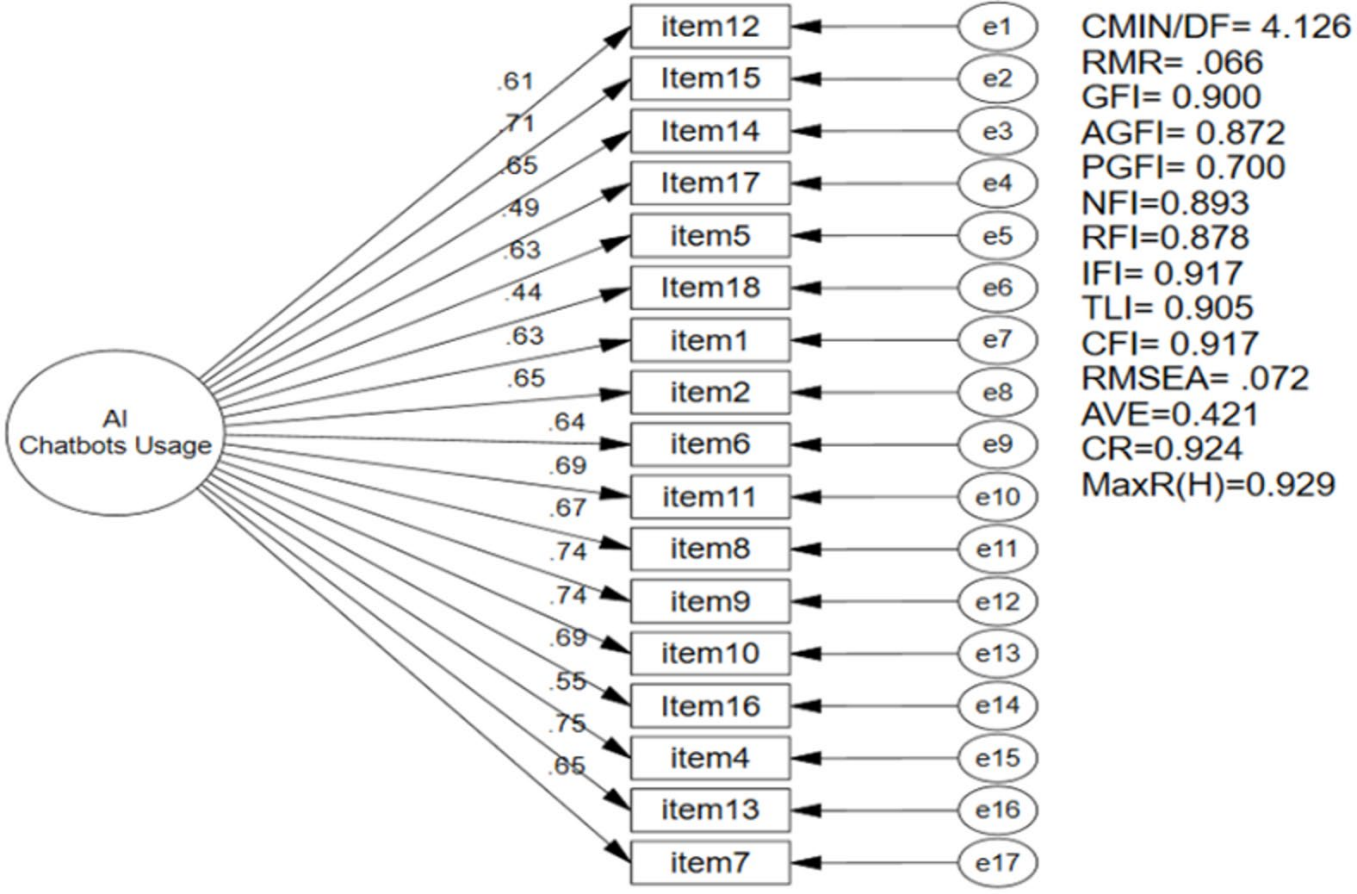
CFA path diagram for unidimensional model.

CFA was conducted using a separate sample to test both the unidimensional and multidimensional models identified in the exploratory phase. The multidimensional four-factor model (Fig. 1) demonstrated superior fit across all examined indices compared to the unidimensional model (Fig. 2). The chi-square difference test revealed a significant improvement in model fit (Δχ² = 238.852), strongly favoring the multidimensional structure.

Specific fit indices for the multidimensional model indicated excellent model fit: χ²/df = 1.704, GFI = 0.957, AGFI = 0.945, CFI = 0.979, RMSEA = 0.034, NFI = 0.951, RFI = 0.944, IFI = 0.979, and TLI = 0.976. All indices exceeded conventional thresholds for good model fit, with RMSEA below 0.05 and CFI, NFI, IFI, and TLI all above 0.95. In contrast, the unidimensional model showed poorer fit across all indices, with χ²/df = 4.126, GFI = 0.900, AGFI = 0.872, CFI = 0.917, RMSEA = 0.072, supporting the rejection of the single-factor solution.

The multidimensional model demonstrated superior parsimony as evidenced by lower information criteria values (AIC = 336.168, BIC = 520.769) compared to the unidimensional model (AIC = 559.020, BIC = 708.459). A comprehensive comparison of model fit indices is presented in Table 2. Convergent validity was supported by an average variance extracted (AVE) of 0.717 and composite reliability (CR) of 0.909 for the multidimensional model, both exceeding recommended thresholds of 0.50 and 0.70, respectively.

**Table 2:** Model fit indices comparison between unidimensional and multidimensional models.

Fit Index	Unidimensional Model	Multidimensional Model	Δ	Interpretation
CMIN (χ²)	491.020	252.168	238.852	Multidimensional model significantly better
CMIN/DF	4.126	1.704	2.422	Multidimensional model shows better fit
RMR	0.066	0.042	0.024	Multidimensional model superior
GFI	0.900	0.957	0.057	Multidimensional model optimal
AGFI	0.872	0.945	0.073	Multidimensional model significantly better
CFI	0.917	0.979	0.062	Multidimensional model substantially better
RMSEA	0.072	0.034	0.038	Multidimensional model optimal fit
AIC	559.020	336.168	222.852	Multidimensional model more parsimonious
BIC	708.459	520.769	187.690	Multidimensional model superior
NFI	0.893	0.951	0.058	Multidimensional model optimal
RFI	0.878	0.944	0.066	Multidimensional model optimal
IFI	0.917	0.979	0.062	Multidimensional model optimal
TLI	0.905	0.976	0.071	Multidimensional model significantly better

### Reliability analysis

Internal consistency reliability was assessed for each factor in the multidimensional model using multiple reliability coefficients (Table 3). The Ease of Use factor demonstrated excellent reliability (McDonald’s ω = 0.893, Cronbach’s α = 0.893, Guttman’s λ₂ = 0.893). The Perceived Usefulness factor showed good reliability (McDonald’s ω = 0.799, Cronbach’s α = 0.799, Guttman’s λ₂ = 0.800), as did the Trust factor (McDonald’s ω = 0.804, Cronbach’s α = 0.804, Guttman’s λ₂ = 0.804). The Accessibility factor exhibited acceptable reliability (McDonald’s ω = 0.734, Cronbach’s α = 0.731, Guttman’s λ₂ = 0.732). The total scale demonstrated excellent internal consistency (McDonald’s ω = 0.927, Cronbach’s α = 0.927, Guttman’s λ₂ = 0.928).

**Table 3:** Reliability coefficients for AI chatbots usage scale factors.

Variable	McDonald’s ω	Cronbach’s α	Guttman’s λ2
**Ease of Use**	0.893	0.893	0.893
**Perceived Usefulness**	0.799	0.799	0.800
**Trust**	0.804	0.804	0.804
**Accessibility**	0.734	0.731	0.732
**Total Score**	0.927	0.927	0.928

### Internal consistency and convergent validity

Correlation analyses revealed significant positive relationships among all factors, supporting the theoretical coherence of the scale (Table 4). The Ease of Use factor showed strong correlations with Perceived Usefulness (*r* = .643, *p* < .01) and Trust (*r* = .702, *p* < .01), and a moderate correlation with Accessibility (*r* = .522, *p* < .01). Perceived Usefulness demonstrated strong correlations with Trust (*r* = .649, *p* < .01) and a moderate correlation with Accessibility (*r* = .431, *p* < .01). Trust and Accessibility factors showed a moderate correlation (*r* = .462, *p* < .01). All factors demonstrated strong correlations with the total scale score, ranging from 0.673 to 0.926, indicating good convergent validity while maintaining sufficient discriminant validity between factors.

To formally evaluate discriminant validity, the Fornell-Larcker criterion was applied by comparing the square root of each factor’s AVE against its correlations with other factors (Table 4). The square root of AVE for each factor exceeded the inter-factor correlations (Ease of Use = 0.847, Perceived Usefulness = 0.796, Trust = 0.801, Accessibility = 0.756), satisfying the Fornell-Larcker criterion and confirming that each factor shares more variance with its own indicators than with other factors. Additionally, all inter-factor correlations (0.431 to 0.702) remained below the recommended HTMT threshold of 0.85, providing further evidence of adequate discriminant validity between the four constructs.

**Table 4:** Fornell-Larcker discriminant validity matrix.

Variable	1	2	3	4	5
**1. Ease of Use**	.**847**				
**2. Perceived Usefulness**	0.643^**^	**0.796**			
**3. Trust**	0.702^**^	0.649^**^	.**801**		
**4. Accessibility**	0.522^**^	0.431^**^	0.462^**^	.**756**	
**5. Total AI Chatbots Usage**	0.926^**^	0.816^**^	0.846^**^	0.673^**^	0

Note: Diagonal values (bold) = √AVE; off-diagonal values = inter-factor correlations; ^**^p < .01.

Overall, the results provide comprehensive support for the psychometric validity of the AI Chatbots Usage Scale as a multidimensional instrument. The four-factor structure (Ease of Use, Perceived Usefulness, Trust, and Accessibility) demonstrated superior fit compared to a unidimensional model, with excellent reliability coefficients and appropriate convergent validity. These findings establish the scale as a reliable tool for measuring university students’ AI chatbot usage patterns.

## Discussion

The four-factor structure identified in this study demonstrates remarkable consistency with established technology acceptance frameworks, particularly the TAM and the UTAUT. The emergence of Ease of Use and Perceived Usefulness as distinct factors directly mirrors TAM’s foundational constructs, which have been consistently validated as primary predictors of technology adoption across numerous educational contexts^38,53^. This alignment is particularly significant given that these constructs form the theoretical backbone of technology acceptance research and have been empirically demonstrated to predict behavioral intentions in AI chatbot adoption studies^54,55^. The robust factor loadings observed for these dimensions (ranging from 0.548 to 0.722) further corroborate previous findings that ease of use and perceived usefulness remain central determinants of student technology acceptance, even in the context of emerging AI technologies.

The identification of Trust and Accessibility as additional factors reflects the evolving nature of technology acceptance frameworks, particularly in AI-driven educational contexts. Trust has emerged as a critical extension to traditional acceptance models when applied to chatbot technologies, with recent studies consistently demonstrating its significant influence on behavioral intention and, in one case, actual usage^16,56^. This finding aligns with UTAUT2’s recognition of trust as a moderating factor in technology adoption, particularly relevant for AI systems where users must rely on algorithmic decision-making processes^16,17^. Similarly, the Accessibility factor corresponds closely to UTAUT’s “facilitating conditions” construct, which encompasses technical support, infrastructure, and system compatibility—elements that have proven essential for sustained educational technology adoption^21^. The moderate to strong intercorrelations observed between all factors (*r* = .431 to 0.702) suggest a theoretically coherent model that extends traditional acceptance frameworks while maintaining conceptual integrity with established constructs.

The psychometric properties of the AI Chatbots Usage Scale demonstrate robust alignment with established educational technology measurement instruments in higher education contexts. The scale’s excellent internal consistency reliability, with McDonald’s ω and Cronbach’s α values ranging from 0.731 to 0.928, compares favorably to established educational technology scales that typically report reliability coefficients between 0.57 and 0.93^47–49^. The CFA results, showing excellent model fit indices (CFI = 0.979, RMSEA = 0.034), are consistent with or exceed the psychometric standards typically reported in educational technology scale validation studies^17,39^. This convergence suggests that AI chatbot usage measurement demonstrates promising psychometric properties that warrant further validation across more diverse samples, with the four-factor structure explaining 42.48% of total variance—a proportion that aligns with variance explained in comparable edtech instruments.

The validation methodology employed in this study mirrors best practices established in educational technology measurement research, utilizing the two-sample approach recommended for robust scale development. The sample sizes of 374 for exploratory analysis and 599 for confirmatory analysis exceed the minimum requirements suggested for factor analysis, consistent with recommended psychometric practices for robust scale development and validation^57,58^. The use of both EFA and CFA on separate samples, coupled with multiple reliability assessment methods, reflects comprehensive validation procedures consistent with current methodological guidance for scale development. However, the current study shares common limitations with other educational technology scales, including reliance on convenience sampling from a single institution and cross-sectional design, which may affect generalizability.

The practical implications of this validated scale extend beyond mere measurement, offering preliminary insights that may inform educational practice and institutional decision-making within similar contexts. Educational institutions can utilize this instrument to systematically assess student engagement with AI chatbot technologies, enabling evidence-based improvements to chatbot implementations and support services. The four-factor structure provides administrators with specific dimensions to target when designing chatbot systems, suggesting that successful implementation requires attention to technical usability, perceived educational value, trust-building mechanisms, and accessibility features. This multidimensional understanding can inform strategic planning for AI integration, helping institutions allocate resources effectively across these critical areas to maximize student adoption and educational impact.

The theoretical contributions of this study advance our understanding of technology acceptance in contemporary educational environments characterized by rapid AI adoption. By validating a comprehensive measurement tool specifically designed for AI chatbot usage, this research provides empirical support for extending traditional technology acceptance models to accommodate the unique characteristics of artificial intelligence in educational settings. The identification of Trust and Accessibility as distinct factors alongside traditional TAM constructs suggests that AI technologies may require more nuanced acceptance models that account for algorithmic decision-making and infrastructure dependencies. This theoretical advancement contributes to the broader literature on educational technology adoption while providing a foundation for future research examining the complex relationships between student characteristics, institutional factors, and AI chatbot usage patterns.

While this study provides valuable insights into AI chatbot usage measurement, several limitations warrant acknowledgment. The reliance on convenience sampling from a single educational institution may limit generalizability, particularly to contexts with different technological infrastructures, cultural backgrounds, or student demographics. The cross-sectional design precludes examination of causal relationships and temporal stability. The study focused exclusively on the Faculty of Education, potentially limiting applicability to other disciplines. Additionally, this study is limited to scale validation (EFA/CFA) and does not test structural relationships, mediating effects, or predictive pathways among constructs; future research using SEM or PLS-SEM is needed to explore the explanatory potential of the validated scale. The self-report nature of the instrument may also introduce response bias.

Future research opportunities emerge from both the validated instrument and the theoretical framework established in this study. Longitudinal investigations utilizing this scale could examine how AI chatbot usage patterns evolve over time and across different educational contexts, providing insights into sustained adoption and long-term learning outcomes. Cross-cultural validation studies would enhance the instrument’s applicability across diverse international educational settings, particularly given the global expansion of AI chatbot implementation in higher education. Additionally, researchers could employ this scale to investigate the relationships between AI chatbot usage patterns and academic performance, student satisfaction, and learning outcomes, contributing to evidence-based practices for educational AI integration. The multidimensional nature of the scale also enables examination of differential usage patterns across demographic groups, academic disciplines, and technological proficiency levels, supporting more personalized and equitable approaches to AI chatbot implementation in educational institutions.

## Conclusion

The development and validation of the AI Chatbots Usage Scale represents a significant advancement in educational technology measurement, providing researchers and practitioners with a psychometrically robust instrument for assessing student perceptions and acceptance of AI chatbot technologies in higher education. The four-factor structure encompassing Ease of Use, Perceived Usefulness, Trust, and Accessibility offers a theoretically grounded and empirically validated framework for understanding the multidimensional nature of AI chatbot adoption. The excellent psychometric properties demonstrated across multiple reliability and validity indicators suggest the scale holds promise as a measurement tool pending validation across more diverse and representative samples. As educational institutions continue to integrate AI technologies into their pedagogical and administrative frameworks, this validated measurement instrument provides essential support for evidence-based decision-making and continuous improvement of AI chatbot implementations. The scale’s alignment with established technology acceptance theories, combined with its extension to address AI-specific considerations, positions it as a valuable contribution to the evolving landscape of educational technology research and practice.

## Data Availability

The datasets generated and analyzed during the current study are available from the corresponding author upon reasonable request.

## Abbreviations

AGFI: Adjusted Goodness of Fit Index
AI: Artificial Intelligence
AIC: Akaike information criterion
AMOS: Analysis of Moment Structures
AVE: Average Variance Extracted
BIC: Bayesian information criterion
CFA: Confirmatory Factor Analysis
CFI: Comparative Fit Index
CMIN: Chi-square
CMIN/DF: Chi-square/Degrees of Freedom
CR: Composite Reliability
DF: Degrees of freedom
ECM: Expectation Confirmation Model
EFA: Exploratory Factor Analysis
GFI: Goodness of fit index
GLB: Greatest Lower Bound
IFI: Incremental fit index
KMO: Kaiser-Meyer-Olkin
MaxR(H): Maximum Reliability coefficient
NFI: Normed Fit Index
RFI: Relative fit index
RMR: Root mean square residual
RMSEA: Root Mean Square Error of Approximation
SPSS: Statistical Package for Social Sciences
TAM: Technology Acceptance Model
TLI: Tucker-Lewis index. Better values are highlighted in green
UTAUT: Unified Theory of Acceptance and Use of Technology
